# Upper extremities and spinal musculoskeletal disorders and risk factors in students using computers 

**Published:** 2014

**Authors:** Bilge Basakci Calik, Nesrin Yagci, Suleyman Gursoy, Mehmet Zencir

**Affiliations:** 1 Bilge Basakci Calik, PT, PhD, Assistant Professor, School of Physical Therapy and Rehabilitation, Pamukkale University, Denizli, Turkey.; 2 Nesrin Yagci, PT, PhD, Associate Professor, School of Physical Therapy and Rehabilitation, Pamukkale University, Denizli, Turkey.; 3 Suleyman Gursoy, PT, PhD, Assistant Professor, School of Physical Therapy and Rehabilitation, Pamukkale University, Denizli, Turkey.; 4 Mehmet Zencir, MD, Professor, Department of Public Health, Pamukkale University, Faculty of Medicine, Denizli, Turkey. School of Physical Therapy and Rehabilitation, Pamukkale University, Denizli, Turkey.

**Keywords:** Musculoskeletal discomfort, computer usage, students

## Abstract

***Objective:*** To examine the effects of computer usage on the musculoskeletal system discomforts (MSD) of Turkish university students, the possible risk factors and study implications (SI).

***Methods:*** The study comprised a total of 871 students. Demographic information was recorded and the Student Specific Cornell Musculoskeletal Discomfort Questionnaire (SsCMDQ) was used to evaluate musculoskeletal system discomforts.

***Results:*** The neck, lower back and upper back areas were determined to be the most affected areas and percentages for SI were 21.6%, 19.3% and 16.3% respectively. Significant differences were found to be daily computer usage time for the lower back, total usage time for the neck, being female and below the age of 21 years (p<0.05) had an increased risk.

***Conclusions:*** The neck, lower back and upper back areas were found to be the most affected areas due to computer usage in university students. Risk factors for MSD were seen to be daily and total computer usage time, female gender and age below 21 years and these were deemed to cause study interference

## INTRODUCTION

Musculoskeletal system discomfort (MSD) is one of the major health problems of modern society, which is common throughout the world.^1^^-^^3^ Computer usage creates a risk of musculoskeletal system discomfort especially for the upper extremities.^4^^-^^6^ In professional life, the upper body, neck, lower back and hands are frequently affected due to computer usage.^7^

There is a higher rate of computer usage among students than adults.^8^^,^^9^ Noack Cooper et al.^10^ stated that students use computers with inappropriate posture and accordingly they feel discomfort in one or more body parts. As university students spend a great deal of time sitting or working, usually with inappropriate posture, it is possible that they may frequently experience musculoskeletal discomforts 

Despite the fact that there is an increasing number of students using computers in the current age of information technology, few studies have been conducted to evaluate students’ computer usage and their ergonomic view^6^^,^^10^^,^^11^ Katz et al.^12^ stated that concrete difficulties while doing work with computer or during personal activities cause musculoskeletal discomforts for students.

Studies have examined musculoskeletal discomfort caused by computer usage prevalence but there has been less examination of the effect of school activities and possible risk factors. Therefore, this study created the hypotheses mentioned below to answer these questions;

1. Computer usage frequently causes musculoskeletal discomfort in university students.
2. An increase in computer usage time increases musculoskeletal system discomforts.
3. Musculoskeletal system problems caused by computer usage are affected by physical factors such as gender and age.
4. Musculoskeletal system problems caused by computer usage affect the education of university students.

## METHODS

For this cross-sectional study which examined musculoskeletal system problems of university students caused by computer usage, 871 students (507 female, 364 male) from different departments of Pamukkale University, Denizli, Turkey were included. Exclusion criteria were pain caused by a chronic disease (cancer or a neuropathic ache), diagnosed rheumatic disease and physical trauma in the previous 3 months. Sociodemographic factors of age, gender, height, weight, body mass index, educational status, and residence were recorded. In addition, the participants were questioned about their computer usage. Approval for the study was granted by the Ethics Committee of Clinical Research, Pamukkale University (B.30.2.PAU.0.20.05.09/13).

The Cornell Musculoskeletal Discomfort Questionnaire (CMDQ), developed by Cornell University, was applied in the study. The reliability and validity of this questionnaire for the Turkish population (T- CMDQ) was tested in 2008 by Erdinc et al. The student-specific CMDQ (SsCMDQ) is a sub type of T-CMDQ, which evaluates pain or discomfort frequency in 11 different parts of the body within the previous 7 days, the level of this pain and whether or not the pain affects the ability to study. Data related to the upper extremities and the spine were collected for this study. Scores were calculated according to the method given below.^13^^,^^14^

***Statistical analysis*** ***: ***The results were analyzed using the Statistical Package for Social Science (SPSS, Chicago) version 16.0 To evaluate socio-demographic variables, descriptive statistical methods were used. Descriptive statistics were stated as mean ± standard deviation (SD) and frequencies (count and percentage). The Chi square test was applied to examine the effect of daily computer usage, years of computer usage, gender and age on discomforts felt in the neck and back. Spearman Correlation Analysis was used to examine the correlation between neck, upper back and lower back scores with computer usage time. The Mann-Whitney U test was used to compare computer usage time with gender and age.

## RESULTS

The study comprised 507 female and 364 male students with a mean age of 21.3±1.8 years. Demographic data are shown in Table-I.

***Student Specific Cornell MS Discomfort Questionnaire (SsCMDQ) results:*** A detailed examination was made of the frequency of musculoskeletal system discomforts, the level, the percentage of effect on lessons and the average scores for the upper extremities and the spine according to SsCMDQ (Table-II). According to the scores of the SsCMDQ, the areas most affected were the neck (47.3%), lower back (43.7%) and upper back (37.8%). When study interference (SI) was examined according to SsCMDQ, the percentages of neck, lower back and upper back were 21.6%, 19.3% and 16.3% respectively. As the weighted scores for these most often affected areas of neck, upper back and lower back were high, detailed examination of these areas was considered (Table-II).

***Computer Usage Time and MSD Results: ***A statistically significant correlation was determined between increased daily computer usage time and discomfort in the lower back area and between increased total computer usage time and discomfort in the neck (p<0.05) (Table-III). No statistically significant correlation was found between daily computer usage time and regional scores or total scores for all three areas. However, a significant and positive relationship was found between total computer usage time and discomfort in the neck area (r=0.068) (p<0.05) (Table-IV).

***Personal Risk Factors; Gender:*** A higher level of discomfort in these three areas was felt by female students than male students and this difference was statistically significant (p<0.05).

***Age:*** Students were divided into two groups of below 21 years of age and above 21 years. The students aged below 21 years reported higher levels of MSD and statistically this difference was significant for the neck and lower back (p<0.05), (Table-III).

***Comparison of Total Scores with Risk Factors: ***In the comparison of total scores and risk factors, computer usage time, gender and age were found to be significant (p<0.05), but daily computer usage time was not found to be significant (Table-V).

## DISCUSSION

This study was conducted to examine the musculoskeletal system problems created by personal and computer-related risk factors. To achieve this, the effects of the computer usage of university students on MSD and the effects on university study were investigated.

**Table-I:** Demographic data of the participants

**Gender** Females Males	n (%) 507 (58.2) 364 (41.8)
**Age**	
Range Median	17-30 years 21 years
**BMI **	
Range Mean (SD)	15.57-40.00 kg/m^2^ 22.3 (3.07) kg/m^2^
**Education**	n (%)
Business- Economics Faculty Engineering Faculty Education Faculty Science-Literature Faculty Academies Faculty of Medicine	237 (27.2) 223 (25.6) 149 (17.1) 140 (16.1) 93 (10.6) 29 (3.3)
**Type of computer** Deskop Laptop Both	n (%) 96 (11.1) 583 (66.9) 192 (22.0)
**Hours of computer use per day** 0-4 h/day 4-8 h/day 8-12 h/day	n (%) 690 (79.2) 167 (19.2) 14 (1.6)
**Total computer use (year)** 0-4 years 4-8 years 8-12 years 12-16 years	n (%) 261 (30.0) 356 (40.9) 208 (23.9) 46 (5.3)
**Causes for interference** Relaxation Walk Sleep Exercise	n (%) 395 (45.5) 176 (20.2) 55 (6.3) 38 (4.4)

**Table-II:** **Student-** **specific** **,** **Musculoskeletal Discomfort Questionnaire **.

**Body Regions**	**Frequency**					**Severity**			**Study interference**			**Weighted scores** **Mean** **±** **SD**
	**Never** **n (%)**	**1-2 times last week** **n (%)**	**3-4 times last week** **n (%)**	**Once every day** **n (%)**	**Several Times every day n (%)**	**Slightly** **n (%)**	**Moderately** **n (%)**	**Severely** **n (%)**	**Not at all** **n (%)**	**Slightly** **n (%)**	**Sub-** **stantially** **n (%)**	
**Neck**	459(52.7)	240(27.6)	101(11.6)	34(3.9)	37(4.2)	185(21.2)	182(20.9)	23(2.6)	683(78.4)	167(19.2)	21(2.4)	4.60±11.76
**Right shoulder**	630(72.3)	133(15.3)	59(6.8)	22(2.5)	27(3.1)	119(13.7)	98(11.3)	20(2.3)	758(87.0)	98(11.3)	15(1.7)	3.00±10.23
**Left** **shoulder**	664(76.2)	113(13.0)	55(6.3)	18(2.1)	21(2.4)	109(12.5)	72(8.3)	23(2.6)	766(87.9)	94(10.8)	11(1.3)	2.51±8.92
**Upper Back**	542(62.2)	161(18.5)	104(11.9)	37(4.2)	27(3.1)	155(17.8)	134(15.4)	27(3.1)	729(83.7)	118(13.5)	24(2.8)	4.01±11.27
**Right upper arm**	802(92.1)	47(5.4)	11(1.3)	6(0.7)	5(0.6)	65(5.6)	20(2.3)	8(0.9)	838(96.2)	26(3.0)	7(0.8)	0.64±4.27
**Left upper arm**	810(93)	38(4.4)	17(2.0)	4(0.5)	2(0.2)	49(5.6)	23(2.6)	5(0.6)	842(96.7)	25(2.9)	4(0.5)	0.46±3.08
**Lower back**	490(56.3)	213(24.5)	100(11.5)	37(4.2)	31(3.6)	162(18.6)	163(18.7)	31(3.6)	703(80.7)	141(16.2)	27 (3.1)	4.26±10.81
**Right forearm**	825(94.7)	31(3.6)	6(0.7)	5(0.6)	4(0.5)	45(5.2)	21(2.4)	3(0.3)	835(80.7)	30(3.4)	6 (0.7)	0.44±3.01
**Left forearm**	834(95.8)	24(2.8)	6(0.7)	6(0.7)	1(0.1)	45(5.2)	15(1.7)	2(0.2)	844(96.9)	22(2.5)	5 (0.6)	0.27±2.02
**Right wrist**	766(87.9)	67(7.7)	21(2.4)	9(1.0)	8(0.9)	70(8.0)	41(1.7)	8(0.9)	821(94.3)	40(4.6)	10 (1.1)	1.14±6.67
**Left wrist**	803(92.2)	48(5.5)	7(0.8)	11(1.3)	2(0.2)	57(6.5)	27(3.1)	3(0.3)	838(96.2)	28(3.2)	5 (0.6)	0.46±3.03

**Table-III:** Associations of individual and computer related risk factors with the occurrence of musculoskeletal discomfort of the spine.

		**MSD during past week**			
		**n**	**Neck Yes (%)**	**Lower Back Yes (%)**	**Upper Back Yes (%)**
**Gender**	Female Male p value*	507 364	295(58.2) 117(32.1) **0.000**	242(47.7) 139(38.2) **0.005**	223(44) 106(29.1) **0.000**
**Age**	≤21 >21 p value*	483 388	255(52.8) 157(40.5) **0.000**	230 (47.6) 151(38.9) **0.010**	194(40.2) 135(34.8) 0.104
**Hours of computer use per day**	0-4 h/day 4-8 h/day 8-12 h/day p value*	693 167 14	337 (48.8) 70 (41.9) 5(35.7) 0.187	314(45.5) 55 (32.9) 12 (85.7) **0.000**	266(38.6) 58 (34.7) 5 (35.7) 0.650
**Total computer use (years)**	0-4 years 4-8 years 8-12 years 12-16 years p value*	261 356 208 46	118 (45.2) 160 (44.9) 103 (49.5) 31 (67.4) **0.028**	125 (47.9) 143 (43.02) 91(43.8) 22 (47.8) 0.263	101 (38.7) 130 (36.5) 77 (37.0) 21 (45.7) 0.660

* Chi-square test.

**Table-IV:** Associations of weighted scores with regions, overall spine musculoskeletal discomfort status with computer usage time.

	**Hours of computer use per day**		**Total computer use (years)**	
	r	p*	r	p*
**Weighted scores with regions**				
Neck Lower back Upper Back	0.007 0.031 0.007	0.829 0.358 0.829	**0.068** 0.003 0.018	**0.043** 0.934 0.507
**Total weighted scores**	0.033	0.33	**0.070**	**0.039**

* Spearman’s Correlation Analysis.

**Table-V:** Associations of individual and computer related risk factors with overall spine musculoskeletal discomfort status.

**Individual and computer related factors**	**Total weighted scores**		
	**n**	**Mean (SD)**	**P**
**Gender** *			
Female Male	507 364	30.38 (53.91) 12.28(27.51)	**0.000**
**Age** *			
≤20 >20	483 388	25.18 (51.43) 19.88 (37.14)	**0.017**
**Hours of computer use per day** *			
≤3 h/day > 3 h/day	569 302	22.33 (45.93) 23.74 (45.23)	0.663
**Total computer use (years)** *			
≤5 years >5 years	387 484	19.04 (36.82) 25.84 (51.51)	**0.029**

* Mann-Whitney U

This study showed that the MSD prevalence of the neck, lower back and upper back areas were highest in SI ratio average WS. When general prevalence was examined, it was seen that51.8 % of students experienced MSD in at least one part of their body while using a computer, which confirms the first hypothesis. In previous studies, it has been stated that MSD in students due to computer usage mostly occurs in the upper extremities and the spine.^6^^,^^10^^,^^11^^,^^15^^-^^20^

There have been some studies related to the daily and weekly computer usage of university students.^15^^,^^21^ However, those studies have reported the difficulty of calculating time.^22^ Peer and Gibney^23^ found daily computer usage to be 2.9 hours and Menendez et al.^19^ reported it as 3.2 hours. In the current study, daily computer usage was found to be 3.1 hours and weekly computer usage, 21.5 hours, which conforms with literature. The results of the current study demonstrated that of the 3 body parts most affected by computer usage, only the lower back had a risk while the neck and upper back areas of the body had no such risks. Despite the fact that previous studies showed that as computer usage time increases, so the neck and upper extremities are affected, in the current study, the frequency of lower back discomfort was observed to significantly increase. Cho et al.^24^ stated that in order to maintain an upright posture, computer users should keep muscle contractions in the body to a minimum level and this may result in lower back pain. Mohseni-Bandpei et al.^25^ reported risk factors for backache to be sitting for a long time, repeated movements and incorrect posture. Similarly, Noack-Cooper et al.^10^ found no relationship between musculoskeletal system discomforts and computer usage time, and it was reported that students shorten their computer usage time when pain is experienced. 

In the current study, a significant increase in neck discomfort was determined with increased total computer usage time. It was also determined that regional scores for total computer usage time were only related with the neck area. This result indicates that long years of computer usage create a risk for the neck area. Ariens et al.^26^ stated that neck flexion posture and sitting posture while using a computer are related with neck pain. Spending 95% of working time sitting and spending more than 70% of a working hour with the neck in 20° flexion increase the risk of neck pain. It is thought that no statistical risk for the lower back and upper back areas is related to being able to support those areas while sitting, but it is hard to set regular adjustments of neck flexion posture. Peer and Gibney^23^, stated that 81% of university students use various techniques (resting, stretching exercises, adaptation of posture or position) to decrease computer usage-related discomforts and this helps relaxation. Jacobs et al.^11^ reported that students using a notebook had lower rates of lower musculoskeletal system discomforts through this use of additional equipment. Hupert et al.^6^ stated that non-measureable factors related to computer usage routines may also affect musculoskeletal system and ergonomic approaches and health briefings could help decrease problems.

After the analysis comparing total scores with risk factors, a significant correlation was determined between total computer usage time and personal risk factors, but no significance was found related to daily computer usage time. This situation indicated that although individuals find solutions for the short-term problems of acute effects of computer usage, they cannot resolve the problems caused by chronic symptoms which develop over many years. These results confirm the second hypothesis of ‘An increase in computer usage time increases musculoskeletal system discomforts’.

When personal risk factors were examined, female gender was seen to be a risk factor. Previous studies, have also stated that females have higher levels of musculoskeletal discomfort related to computer usage and that that in professional life, computer usage creates more musculoskeletal system discomfort risks for women.^6^^,^^10^^,^^20^

When the age group (17-30 ages) of the students involved in the study was determined, the parameter of age was included in the study. The results demonstrated that students aged below 21 years were at greater risk of MSD in the neck and lower back area. This suggests that younger students use computers for non-lesson related activities for a long time with poor posture and they do not make any adjustments to protect themselves. It is also thought that after many years of computer usage, individuals develop adaptations and make ergo therapeutic interventions to reduce discomforts, such as taking a break and correct posture. Previous studies support this view.^10^^,^^11^^,^^17^

In the SsCMDQ, the effect of computer-related pain, ache and discomfort on lessons is questioned. The results of the current study that lessons were affected by discomfort in the areas of neck (21.6%), lower back (19.3%) and upper back (16.3%) at higher rates than those reported in other studies. This supports the final hypothesis. Katz et al.^12^ and Hupert et al.^6^, stated that daily and school-related activities are limited due to computer usage related pain and Menendez et al.^19^ stated that 10% of students experience difficulty doing school activities. The current study evaluated the most affected body parts in detail, which may explain the higher percentages than those reported in literature. However, one of the aims of the study was to draw attention to examining this situation in more detail.

***Limitations and future studies:*** In the current study, a more objective unbiased method than the one used to calculate and determine computer usage time was not available. Another limitation was that the use of additional equipment and environmental conditions were not questioned (height of table and chair used, environmental lighting, temperature, noise). The use of systems and software to monitor computer usage time and movements would be helpful in obtaining more unbiased results in future studies. To give more reliable results, specific groups could be created, such as single gender, those using laptops or desktops and those who have used a computer for more than 5 years with daily usage of more than 4 hours. With increasing computer usage in all societies and the inherent risks of musculoskeletal discomfort, there is a need for more extensive research using unbiased evaluation methods. Ergo therapeutic education approaches towards students to reduce potential problems would also be of benefit.

## CONCLUSIONS

The results of this study have shown that the computer usage of university students mostly causes neck, upper back and lower back discomfort, which has a negative effect on their study program. Daily computer usage affects the lower back area and total usage time has a cumulative effect on the neck area. Female gender and age below 21 years were also observed to be risk factors. 

The importance of correct posture and back support should be emphasised to reduce pain in the lower back area. Appropriate adjustment of the angle of the monitor, frequent breaks and exercises while at the computer will be effective in reducing discomfort in the neck area. With a reduction in discomfort, the student will feel more comfortable, which will have a positive effect on the study program and success rates. As female gender and a young age were seen to be risk factors, future studies aimed at reducing computer usage related musculoskeletal discomfort in universities should focus on this group.
